# Neither, Nor

**DOI:** 10.4269/ajtmh.26-0436

**Published:** 2026-09-24

**Authors:** B. Jason Brotherton

**Affiliations:** Department of Global and Border Health, University of Arizona, Tucson, Arizona, USA

“Tell me about a successful quality improvement project.” This was the prompt I was given in a fellowship interview. Sure, I had some polished examples I could have shared, but instead what I shared was the opposite.

Several years earlier, I moved to rural Kenya to teach in a new residency program for African physicians. At that time the ink wasn’t even fully dry on my residency diploma, but I was full of ideas and painfully unaware of my limitations. After months of trying to catch up clinically (so much for being a “teacher”), I turned my attention to what I thought was low-hanging fruit.

Many of the children admitted to our hospital had illnesses that were only made worse by their underlying malnutrition. This particular hospital had a nutritionist who accompanied us on rounds with hospitalized patients, and it had an outpatient nutrition clinic for follow-up. Improving our screening for malnutrition was seemingly straightforward, and the impact of diagnosing and referring early could create better outcomes. Win-win.

Off to our medical records department I went to get an idea of how good we were at documenting *Z*-scores and mid-upper arm circumference (MUAC) measurements. To try to account for rainy season and school schedules, an eager visiting medical student and I went through a year’s worth of charts. We indeed found that the hypothesis appeared true; our screening was inconsistent, diagnoses were rare, and referrals almost nonexistent. Confident we had identified the gap, we decided it was time to move on to implementation.

Gathering the interns and medical officers, we reviewed the importance of measuring *Z-*scores and MUAC, and reiterated the mechanics of how to do those measurements. I returned to the charts a couple of weeks later to find that more screenings had been performed but saw no more diagnoses and no new referrals to the nutrition clinic. Unphased, I went through rinse-and-repeat sessions with our learners. Two more weeks passed, and again there were no new diagnoses and no new referrals.

Nonplussed, I went to see the nutritionist to relay my frustrations, assuming that I would encounter validation and a sympathetic ear. Patiently waiting for my tantrum to come to a close, she finally said, “Daktari, you are asking the wrong question. What is the implication of making this diagnosis for these children? Their mothers?”

I was frozen and confused. Never had I considered asking this question, because I didn’t know I should.

Sensing my ignorance, she explained that diagnosing a child with malnutrition could be a socially devastating act. There could be shame directed toward the mother, judgment from the community, and ongoing implications about caregiving and worthiness. My simple pathway to addressing malnutrition risked publicly labeling families in ways that could follow them for years to come. Shame could not begin to cover the feelings I had in that moment of discovery.

In global work, we often read stories of outsiders coming in with their own agenda, not asking questions (be they good or genuine), and doing what they think the community needs. This situation is my own spin-off of that mistake. It wasn’t that this community didn’t care about malnutrition. Of course they did! Although my priorities and intended outcomes aligned with those of parents and the broader community, my framework failed to account for the cultural and relational realities that shaped how care could safely be delivered. It also didn’t even come close to examining the deeper issue of why malnutrition and food scarcity were present to begin with.

Solutions divorced from cultural context are limited at best and more disastrous than the original “quality improvement” (QI) area being addressed. I am thankful that this failure happened early in my global health career. I learned more from this singular experience than any classroom had ever taught me. When I recounted the situation to the interviewer, I summarized the project as neither enhancing quality nor providing any tangible improvement. However, it was a humbling paradigm shift for me and will forever be the most “successful” QI project: improving my humility, curiosity, accountability, and social intelligence.

